# Relational spirituality and quality of life 2007 to 2017: an integrative research review

**DOI:** 10.1186/s12955-018-0895-x

**Published:** 2018-04-24

**Authors:** Victor Counted, Adam Possamai, Tanya Meade

**Affiliations:** School of Social Sciences and Psychology, Western Sydney University, Sydney, Australia

**Keywords:** Relational spirituality, Quality of life, Integrative research review

## Abstract

**Background:**

Despite the increasing number of evidence-based research on relational spirituality (RS) and quality of life (QoL) in medical-health research, little is known about the links between RS and QoL outcomes and the mechanisms by which RS aspects are functionally tied to QoL.

**Objective:**

To determine how RS is perceived/positioned in relation to QoL, we (a) examined recent available data that identify and appraise the links between RS and QoL; (b) identified themes emerging from the association between RS and QoL, and (c) discussed the implications of the effects of RS on QoL outcomes.

**Methods:**

We conducted an integrative research review of English-language peer-reviewed articles published between 2007 to March 2017 which examined an association between RS and QoL, as identified from a search of three databases: PubMed, PsycINFO, and ScienceDirect.

**Results:**

A total of 20 studies were analysed. Of these, twelve (60%) reported positive association between RS and QoL, three (15%) studies reported inverse associations, whereas five (25%) studies showed evidence of lack of association (with two out of the five studies showing an indirect association). Physical health and psychological functioning were the most researched domains of QoL, and some studies suggest an attachment-based model of RS in the last 10 years of RS and QoL research. Studies conducted with participants with serious illnesses ranging from dementia, cardiac arrest, and breast cancer reported no association between RS and physical health. Our review shows evidence of both the direct and/or indirect effects of RS on QoL as a possible spiritual coping model for complementary alternative health therapy, albeit occurring through several religious-related psychosocial conduits.

**Conclusion and implication:**

RS appears to be associated with health benefits as indicated across QoL domains. General medical practitioners and other healthcare agencies could benefit from the understanding that a spiritual coping model could aid their patients, and therefore their clinical practices, in the healing process.

## Background

There is a growing body of research that promotes spirituality as an internal locus of control in terms of regulating negative emotional states [1, 2]. These studies assert spiritual activities as helpful means of attaining self-transcendence and coping with life stressors. Chaney and Southwick [3] made such a claim in their book *Resilience*, describing how people overcome life’s challenges by searching for connectedness and seeking comfort in spirituality based on what is transcendent. Spirituality differs in many ways from ‘religiosity/religiousness’. The former emphasizes the personal character of spiritual transcendence in terms of the changing, developing constitutive trait and dynamic attribute one has from cradle to grave [4], whereas the latter is considered in terms of the institutionalised character of religious beliefs. In this paper, we refer to relational spirituality (RS) as the former—as an important platform for personal development that draws people to forge meaning in life through spiritual transcendence [5]. Piedmont [6] ascribes a special role to RS with regards to spiritual transcendence, arguing that it should be considered as the sixth domain of personality since it represents a broad-based motivational domain and language term (e.g., universality, prayer fulfilment, and connectedness) that capture adaptively important psychological qualities that are comparable to The Big Five personality: Neuroticism, extraversion, openness to experience, conscientiousness, and agreeableness. Several other authors have weighed in on the concept of spirituality and provided different definitions. For example, as “the personal quest for understanding answers to ultimate questions about life” [7], “an organised system of beliefs and symbols designed to facilitate closeness to the sacred” [8], an aspect of quality of life [9, 10], “as subjective belief systems that incorporate self-awareness and reference to a transcendence dimension” ([11]: 288), as a perceived relationship with a ‘divine attachment figure’ [12], and the “unique window into attachment processes in adulthood” ([13]: 917).

In examining the above definitions, it seems that RS is mostly discussed within two categories: (1) as a cognitive appraisal of existential issues and (2) as the experiential knowledge of the divine [14]. The former recognises RS in connection to the cognitive appraisal of stressors and interpersonal struggles when the individual is in a relationship with the sacred and often referred to as the ‘head-knowledge of God’ [15]. The latter focuses more on the ‘heart-knowledge of God’ [15] in its application of attachment theory and developmental psychology. Both perspectives of RS draw largely from a relational perspective [16], and will be used for guiding this review in terms of the conceptualisation of RS as implicit relational and experiential representations of the sacred that are essential to faith development, spiritual transcendence, and appraising life stressors and QoL.

QoL is used in health-related research to study life satisfaction and the extent to which an individual evaluates their holistic life experience from a subjective point of view [67]. It is a broad-ranging concept “affected in a complex way by the person’s physical health, psychological state, level of independence, social relationships, and their relationship to salient features of their environment” ([75], p.1404), and covering several aspects of individual needs satisfaction that constitute aspects of life that contribute to overall experience.

QoL has much to do with individual satisfaction with key areas of life that enhance a general sense of physical and emotional well-being. Research shows that this concept is applicable for adolescents [17], women [18], migrants [19, 20], older adults [21], religious people [22], non-religious groups [23], among others. Studies have also shown that QoL has much broader implication for health care [24] and life satisfaction [25]. As a context-specific construct [26], measures of QoL are used to assess the individual evaluation of their health-related outcomes, and in recent cases, modified for assessing disease-specific QoL outcomes. These include, HIV/AIDS-targeted QoL [27], geriatric quality of life-dementia (GQOL-D) [28], QoL—alzheimer disease scale (e.g., [29]), and heart-disease specific QoL scale (e.g., [30]).

Researchers recognise QoL as a multidimensional concept in terms of its subjective, objective, and culture/spirituality components, while at the same time identifying the relationship between these aspects (e.g., [9, 25, 31, 32]). Marshall [33] and Rule [34] mention income, education, and housing as examples of objective aspects of QoL. Collinge et al. [35], along with O’Connell and Skevington [36], spirituality’ as/spirituality’ as an important aspect of QoL.

The subjective satisfaction aspect is the most researched aspect in QoL literature, as it mostly assesses physical health, psychological health, social relationship health, and environmental health (e.g., [31, 35]). While QoL is likely to be related to individual contexts, as well as to the subjective satisfaction aspects, we argue that the spiritual aspect may be related to beneficial health outcomes and should be positioned as an additional conceptualisation of RS. A similar proposition can be seen in other studies (e.g., [9, 10]), concluding that one’s spiritual well-being is an important aspect of QoL since it influences the subjective domain. The concept of QoL is continuum in nature since it considers overall life satisfaction that is connected with the individual’s physical, mental, environmental, social, and spiritual functioning. Nonetheless, QoL is constantly changing, depending on the lived experience resulting from several factors that control one’s life, especially ones related to self-transcendence and the connection to the sacred [37].

Based on this theoretical and empirical background, RS may be perceived as a relational dynamic and an implicit relational representation (e.g., [16]) that highlights a perspective which seeks to understand the human need for an object of devotion and spiritual functioning, guiding the quest for meaning and attachment to the sacred (e.g., [16, 38, 39]), in terms of its association with QoL. This relational perspective is largely drawn from the works of object relations theorists (e.g., [40–42]), and recently, RS has been theorized from an attachment theoretical perspective [16]. Although the original thesis on attachment proposed by Bowlby [43] largely assumed an evolutionary perspective and its manifestation in parent-child relationships, he also noted that attachment processes may have a broad implication for psychological development throughout a person’s life. Cicirelli [44] studied lifespan attachment developments among older adults, with empirical evidence supporting a strong sense of attachment to God among older adults. Other studies (e.g., [16, 37, 45]) have proposed an idea of a RS that can be interpreted in terms of an attachment-based model, one defined as a perceived relationship with the sacred. Granqvist and Kirkpatrick [13] refer to this as the maturational aspect of attachment development resulting from the increased ability in core specific cognitive tasks such as processing speed (e.g., [46]), voluntary response suppression (e.g., [47]), and working memory (e.g., [48]), which are usually immature in childhood [49].

Several studies (e.g., [13, 16, 50]) have integrated the principles of Bowlby’s [43] attachment theory into the study of religious and spiritual development. Aspects of RS as an attachment-based model of spiritual development have been reported in studies (e.g., [37]) suggesting that attachment relational variables consistently emerge as predictors of various outcome variables that match spiritual and psychological well-being. For example, Augustyn et al. [37] report that RS (through spiritual practices such as prayer, awareness of God, spiritual meaning, and forgiveness) strengthens the link between spiritual well-being and psychological outcomes. Bradshaw and Kent [22] report that RS in terms of an attachment connection to the sacred strengthens the relationship between prayer and psychological outcomes. However, Sloan et al. [51, 52] have criticized empirical studies’ claim of health benefits associated with religiosity, arguing that about 83% of the studies making such claims were methodologically flawed and misrepresented data; thus, there is no empirical basis for asserting that religious involvement is related to positive health outcomes. Miller and Thoresen [53] have argued that the consistency of empirical reports on the effects of spirituality on health-related outcomes point toward salutary effects. Other studies (e.g., [54, 55]) have linked adverse effects to beliefs in a punitive divine figure, extrinsic religiousness, and upholding a religious moral standard.

Recent literature reviews (e.g., [56, 57]) have shown evidence of positive links between spirituality/religiosity and QoL, albeit these studies were mostly limited to organizational religious affiliation among elderly adults in South America (e.g., [56]) and how religious participation correlates to mental disorders (e.g., [57]). These two reviews found empirical support for the association between several aspects of religiosity and health and mental health outcomes. For example, Bonelli and Koenig’s [57] review provides evidence to show that religious involvement is associated with better mental health outcomes in 72.1% of the studies, while Abdala et al. [56] concluded, based on the positive association in 75% of the studies, that the religious affiliation of elderly South Americans is associated with better outcomes of quality of life. However, it seems that the conceptualisation used in these studies differ from that offered in our proposed study in terms of our definitions of RS and QoL. There appears to be no recent review study which appraises RS as (1) a domain of QoL that relates to spiritual functioning, and (2) a relational dynamic in terms of the relationship connection with a symbolic sacred object or figure, either through spiritual transcendence, prayer fulfilment, attachment to God, or other implicit relational conduits. In addition, while much has been done in promoting RS as an aspect of QoL, little is known about the broader perspective regarding the association between RS and QoL in disease-specific medical-health research. With debate around the salutary effects of RS on health outcomes, this paper contributes to the ongoing debate on how RS functioning is associated with perceptions of QoL. This study will thus: (1) review recent available data that identify and appraise the links between RS and QoL; (2) determine how RS is perceived/positioned in relation to QoL; (3) identify emerging themes emerging from the association between RS and QoL and (4) discuss the results and implications of the association between RS and QoL.

## Methods

### Review design

An integrative research review (IR) design is used to appraise the existing literature on RS and QoL. IR is a ‘systematic’ process “restricted to relevant studies that point to new data related to the study goals” ([58]: 12). The choice of an IR allows for synthesizing knowledge from diverse disciplinary sources to better describe a subject [59]. The use of this method to conduct a systematic literature review is especially appropriate for interpreting complex subjects. This is because it deconstructs theoretical and interdisciplinary domains related to such topics in order to formulate new reliable models, research agendas, or a meta index of meanings and definitions [60]. An IR contrasts with meta-analysis in that its aim is to have a comprehensive collection of literature, especially when the subject is in one knowledge domain. An IR is comparative for review of subjects from diverse knowledge domains, as is the case in this review, and consistent with previous review on the subject (i.e., [56]). An IR also has a more flexible and inclusive review design (involving both quantitative and qualitative studies), compared to other types of review methods. It allows for a more purposeful selection and inclusion of diverse data sources and empirical contributions which show the association between RS and QoL in diverse and broad sampling frames. Hence, we have targeted and integrated representative sources of research which have the potential to identify and appraise the links between RS and QoL.

### Data sources

**Fig. 1:**
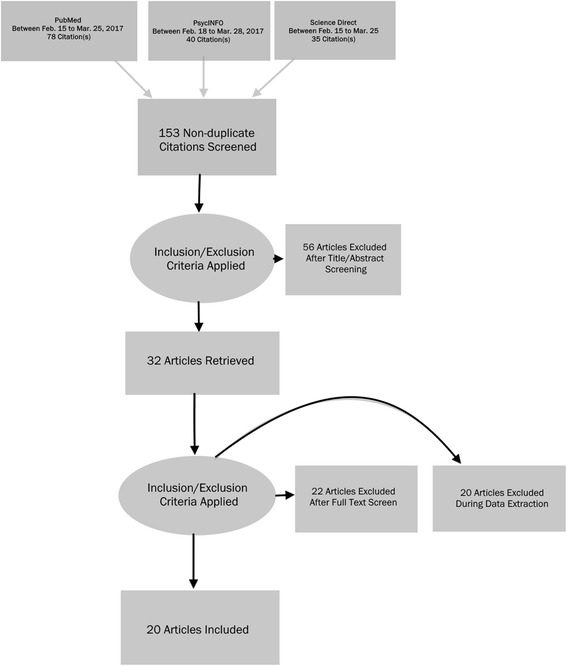
Prisma flow diagram of the study based on literature search from February to March 2017

As shown in Fig. 1, a search was carried out between February and March 2017 using three online databases (i.e., PubMed, PsycINFO, and ScienceDirect) with DeCs^1^ and MeSH^2^ keywords such as ‘religion’ or ‘spirituality’ and ‘quality of life’ or ‘health,’ and later specified using the following keyword strings: ‘spirituality and quality of life’; ‘religion and quality of life’. We chose PubMed, PsycINFO, and ScienceDirect as reliable electronic databases for our literature search because they are leading sources for assessing scientific, technical, and medical research, which have not been used in previous literature reviews, except for Bonelli & Koenig [57] who used PubMed in their literature review search. Abdala et al. [56], on the other hand, used different sources for their literature search, including Virtual Health Library, Latin American and Caribbean Health Sciences Literature (LILACS), Scientific Electronic Library Online (SciELO), Medical Literature Analysis and Retrieval System Online (Medline), and the U.S. National Library of Medicine.

A variety of online database routes were explored in order to ensure that relevant studies related to RS and QoL were retrieved, especially ones that had not been covered in previous reviews. PubMed, PsycINFO, and ScienceDirect were informative in regard to literature reporting on the effects of RS on QoL. Study abstracts bearing the relevant keywords were extracted and read by the first author (see Fig. 1), and the outcome of the studies was reviewed by the second and third authors. Over 185 articles were found in the initial online search, from which 32 articles were selected after reading the abstracts while 153 were rejected due to their lack of clarity in terms of the conceptualisations of RS and QoL offered in the studies. After extensive data screening and full-reading of the 32 selected articles, as of the time of conducting this review (March 2017), 12 studies were rejected for not meeting the selection criteria, leaving 20 for review. Studies were either included or excluded for the review based on the following criteria:

### Inclusion criteria

- Date of publication: 2007 to March 2017.
- English-language peer-reviewed articles with published abstracts on PubMed, PsycINFO, and ScienceDirect.
- Empirical, cross-sectional, longitudinal, quantitative, and qualitative studies.
- Studies reporting on the association between RS and QoL.
- Studies that explored either one or two or all aspects of QoL in terms of its subjective, objective, and spiritual/cultural factors.
- Studies that conceptualised RS as a domain of QoL concerned with spiritual functioning and a relational dynamic that represents attachment to a sacred object or figure.

### Exclusion criteria

- Non-English publications.
- Reviews, theoretical articles, books, chapters, dissertations, conference proceedings, blog articles, and working papers.
- Studies not indexed by either PubMed, PsycINFO, or ScienceDirect.
- Studies published before 2007 and after March 2017.

### Data analysis

Information was collected regarding several study characteristics, including the name of the author(s), country where the study was conducted, sample size, research design, context, and sample characteristics. Data were extracted from studies examining the association between RS and QoL and an IR was conducted following the five-step guideline recommended by Whittemore and Knafl [59]:

I) Recognise the research problem and/or analysis purpose
II) conduct a systematic literature search of recent studies
III) appraise and summarise the quality and results of the selected articles
IV) review selected quality articles to identify possible themes
V) organise the themes and critically analyse them in relation to the research problem.

First, the purpose of our review is to address the claims about RS and QoL outcomes so as to determine how RS is perceived/positioned in relation to QoL and the emerging themes that can help in interpreting the association between RS and QoL. We summarized results from previously conducted studies to appraise the conclusions from all studies relating to the claims of the effects of RS on QoL. As shown in Tables 1,^3^ and 2,^4^ we summarized and compared empirical data in our IR to allow for the achievement of general conclusions about the research problem [58], and analysed results as direct evidence, indirect, positive, and negative associations and lack of associations.

**Table 1:** Articles related to relational spirituality and quality of life for the last 10 years

References	Context & Method	Outcome/Findings
***Idler et al. [62].	A prospective study. Interviews (*n* = 499) from 1982 to 1994 with terminally ill elderly persons in the last year of their lives. Gender (M = 41%, F = 59%) Age range: 65 years and over Mean Age: 74.5 Country: USA	Those who had a sense of religious attachment were more likely to see friends, and they had better QoL, fewer depressive feelings, and were observed by the interviewer to find life more exciting compared with the less religious respondents.
*** Saffari et al. [63].	Cross-sectional survey design (n-362) with Muslim patients undergoing haemodialysis. Gender (M = 46.1%, F = 53.9%) Age range: 20 years and older Mean Age: 57.81 (SD = 9.67) Country: Iran	Spiritual/religious factors were related to QoL and health status. Regression models revealed that demographics, clinical variables, and especially spiritual/religious factors explained about 40% of variance of QoL and nearly 25% of the variance in health status.
# and #* Nagpal et al. [29].	Quantitative interviews and cross-sectional design with 111 Individuals With Dementia (IWD) and their family caregivers from two service-based organizations in the San Francisco Bay Area and Cleveland, Ohio. Age range: 30 to 90+ Mean Age: Caregiver (M = 61.20, SD = 14.00); IWD (M = 76.80, SD = 8.90). Country: USA	After controlling for care-related stress, one’s own religiosity is not significantly related to individuals’ or caregivers’ perceptions of the QoL of individuals with Dementia. However, when modelled for both the individuals and their caregivers, effects of religiosity on perceptions of QoL, caregivers’ religiosity was positively related to the QoL of individuals with Dementia whereas the religiosity of individuals with Dementia was negatively associated with caregivers’ perceptions of IWDs’ QoL.
# Miller et al. [30].	Cross-sectional survey with 44 (dyads) couples between 49 and 73 years of age following a first-time cardiac event. Gender: Patient Males (*n* = 35) and Females (*n* = 9). Age range: Patients (49 to 73 years of age); Spouses (47 and 71 years of age). Mean Age: Spouses (M = 59.1, SD = 12.6); Patients (M = 61.6, SD = 11.8).	The findings suggest that there is no association between dimensions of spirituality and QoL and perception of the patient’s physical self-efficacy following a first-time cardiac event.
# Nguyen et al. [75].	A prospective study. Data was analysed from the 2002 National Health Interview Survey (*n* = 106,000) that covered responses regarding use of complementary therapies reported by older adults aged 55 to 85 and the association with health outcomes assessed in the 2003 US Medical Expenditure Panel Survey (*n* = 1683). Gender: (M = 40.5%, F = 59.5%) Age range: 55 to 85 years of age. Mean Age: (M = 68.5, SD = 0.3) Country: USA.	Even though there was no association between prayer and functional status or QoL, results show that the use of prayer for health was the most common complementary alternative medical therapy reported by those aged 55 to 85 (52.3%), which was more than twice as common as any other category of complementary therapy in term of users of biologically based therapies (20.4%) which predicted better functional status.
***Ai et al. [64].	Quantitative interviews with 294 middle-aged and older patients following open-heart surgery. Gender: (M = 58%, F = 42%) Age range: aged 35 to 89 years of age. Mean Age: (M = 62) Country: USA.	Results show support for adaptive faith-based coping in patients, suggesting that prayer coping was positively associated with cognitive and behavioural changes, as well as perceived social support from family, friends, and significant others at the time of participant’s surgery.
***Calvo et al. [70].	Quantitative interviews with 75 consecutive Amyotrophic Lateral Sclerosis (ALS) patients and their informal caregivers, using tests evaluating religiosity, QoL, satisfaction with life. Gender: Caregivers (26 males and 49 females); Patients (40 males and 35 females) Mean Age: Caregivers (M = 55.8, SD = 12.0); Patients (M = 63.6, SD = 9.2)	Results showed that the QoL of the caregivers of patients with ALS was associated with their private religiousness (i.e. subjective religiosity) whereas their satisfaction with life related to their overall religiosity.
***Kashdan & Nezlek [72].	A 1239 days daily diary study on 87 college students using lagged analysis to examine whether spirituality is causing greater well-being or vice versa. Gender: (M = 23.5%, F = (76.5%) Mean Age: (M = 21.62, SD = 2.36) Country: USA.	There was a significant positive relationship between daily spirituality, self-esteem, meaning in life, and positive affect. Furthermore, present-day spirituality was associated with next day’s meaning in life. There was no evidence shown that supports that meaning in life predicted next day spirituality. Lower positive affect and greater negative emotion on 1 day was associated with greater spirituality on the next day.
***Nolan et al. [65]	Quantitative interviews were conducted with stable outpatients with schizophrenia (*n* = 63), mostly African-Americans (nearly two-thirds of the sample size) living in south-eastern United States to examine the role of religion in coping with their disorder. Gender: (M = 52%, F = (48%) Mean Age: (M = 42.2, SD = 11.6) Country: USA.	Results show that about 68% of the participants were involved in one form of religious activity that involved their religious others. 64% of the participants indicated that being connected to a faith community was important to them. 91% indicated that they were involved in private religious activities that involve praying at least once a day. These kinds of positive religious coping (i.e., religious forgiveness, seeking spiritual support, collaborative religious coping, spiritual connection, religious purification, and benevolent religious reappraisal) were associated with greater QoL (*r* = .28, *p* = .03) and psychological health (b = .72, *p* = .05) whereas negative religious coping (i.e., spiritual discontent, reappraisal of God as punishing, interpersonal religious discontent, reappraisal of demonic powers, and reappraisal of God’s powers) in the form of feeling abandoned by God was associated with worse QoL (*r* = .30, *p* = .02).
#* and # *Bradshaw & Kent* [22]*.*	Longitudinal study. Data collected from nationwide Religion, Aging, and Health Survey from 1024 older Americans. Gender: (M = 38.3%, F = (61.70%) Mean Age: 75.15 Country: USA	Prayer is not directly associated with improvements in psychological well-being. However, when moderated by attachment to God there was a relationship between prayer and psychological well-being. This association was only seen in individuals with secure attachments and not with those who are insecurely attached to God.
# Sorensen et al. [66].	A cross-sectional survey from a sample of 2086 cancer patients and 6258 cancer-free controls from the Nord-Trøndelag Health Study in Norway that took place between 2006 and 2008. Mean Age for cancer patients (Sample), 6.9 years; cancer cases (Sample 2a), 7.7 years; cases of breast, prostate, or colorectal cancer (Sample 2b), 6.3 years. Country: Norway	Spirituality in terms of ‘seeking God’s help’ was associated with lower levels of sexual QoL in the unadjusted model, but when adjusted for other factors (e.g., gender, age, anxiety, neuroticism, extraversion, follow-up time, daily smoking, infrequent exercise, negative outlook, and positive outlook) it did not remain significant for life satisfaction or to QoL measures. There was a lack of association between ‘Seeking God’s Help’ and Life Satisfaction among patients, nor was ‘Seeking God’s Help’ associated with Disease-Specific QoL in patients with breast, prostate, or colorectal cancer.
**Yun et al. [67]*.*	A prospective cohort study of 481 terminally ill Korean cancer patients. 76% (*n* = 466) were interviewed till the time of death. Mean age: Users of complementary alternative medicine (CAM) (58.2 years); Nonusers of CAM (59.0 years). Gender: Users of CAM (Males = 42%, Females = 58%) Nonusers of CAM (Males = 42%, Females = 58%) Country: Korea	Those who used mind-body interventions (e.g. meditation, prayer therapy, music therapy, art therapy, yoga, horticultural therapy) experienced a significant decline in their QoL compared to non-users. Participants using prayer therapy showed a significantly worse survival of insomnia.
**Levin [73].	Data collected from a cross-sectional survey of a random national sample of Jewish participants (*n* = 1287) who are 50 years and older. Mean age: 64.4 years Context: Israeli sample in Europe	Frequency of prayer was inversely related to self-rated health, and positively associated with activity limitation, physical symptoms, and poor physical functioning.
***Moon & Kim [28].	A cross-sectional survey with older Koreans (*n* = 274) 65 and over, living alone in Chuncheon, South Korea. Mean age: (M = 76.76, SD = 6.18) Gender: Females = 82.1%, Males = 17.9%.	There were associations between dimensions of depression, QoL, and spirituality. Spirituality explained the variance on depression and QoL amongst Christians, but did not account for the difference in the Buddhist sample.
***Krumrei et al. [74].	A cross-sectional survey with 208 Jewish men and women. Mean age: M = 42, SD = 12 Gender: 74.5% (Females), 25.5 (Males). Participants were based in U.S. (83%), Canada (7%), Israel (6%), and other countries (4%)	There was a positive correlation between physical health and trust in God (r.14, *p* < .05), and inverse relationship with mistrust in God (−.16, p < .05) and negative religious coping (r.14, p < .05). When adjusted for gender and age, correlation with physical health remained significant, especially in trust in God and physical health.
***Lee et al. [27].	A cross sectional study with 198 persons with HIV/AIDS in urban Philadelphia. Mean age: 44.89 years Gender: 60.5% male (female = 39.5%) Country: USA	Results of the multiple hierarchical analyses reveal that negative religious coping was significantly related to low levels of QoL when adjusted for demographic and clinical variables. Positive religious coping was also significantly associated with positive affect and life satisfaction, but not with overall QoL.
***Currier et al. [68].	A cross-sectional data on 678 military Veterans with posttraumatic stress disorder (PTSD). Mean age: 51.57 years (SD = 9.57 Gender: 94.8% male, 5.2% female Country: USA	When adjusted for demographic risk factors, combat exposure, and severe PTSD symptoms in the structural equation modelling, results revealed that spirituality was significantly associated with forgiveness and QoL. “Higher levels of spiritual functioning were associated with fewer forgiveness problems among these Veterans, and their propensity to forgive self and others was also concurrently linked with QOL” (p.175).
***Canada et al. [69].	Mediation analyses was conducted on data collected from the American Cancer Society’s Study of Cancer Survivors-II (*n* = 8405). Mean age: 63 years Gender: Female (55.1%), Male (44.9%) Country: USA	Results show evidence that faith was strongly associated with meaning and peace in uncontrolled analyses. The mediation analyses show that faith had a significant positive effect on mental functioning (when mediated with greater meaning) and physical functioning (when mediated by both meaning and peace).
***Krause et al. [76].	Data were collected from a nationwide survey with adults (*n* = 1774) from 18 years above. Mean age: 53.1 years (SD = 18.7 years) Gender: Males (38%), Females (62%) Country: USA	The structural equation modelling analyses revealed that those who received spiritual support from members of a faith community experienced stronger benevolent images of God (B = .362, *p* < .001) which influenced QoL. Results also suggest that those who have gratitude to God had more hope about the future (B = .214, p < .001), and hope was associated with better physical health (B = .330, p < .001)
# Rohani et al. [71].	A cross-sectional survey with Iranian women with breast cancer (*n* = 162). Mean age: Breast cancer patients: M = 46.1, SD = 9.8; control group: M = 46.6, SD = 8.4 Country: Iran	Spirituality and positive religious coping was not associated with increases in QoL in Iranian patients.

Note: Showing evidence of: *** positive association between RS and QoL. #* indirect association between RS and QoL. ** negative association between RS and QoL. # lack of association between RS and QoL

Based on Whittemore and Knafl’s [59] second guideline, literature search of recent studies between 2007 to March 2017 was carried out using three online databases (e.g., PubMed, PsycINFO, and ScienceDirect) with the aim of identifying studies that have investigated the links between RS and QoL. Third, to appraise and evaluate the quality and results of selected studies, we presented a clear and synthetic description of study data using Tables 1 and 2. Summarizing the data in tables allowed for the identification of knowledge gaps and pointed out the state of the art of the scientific production that results from selected studies examining the effects of RS on QoL [58]. For this reason, Tables 1 and 2 are drawn as concepts matrix [61] to help highlight study characteristics and summarise key themes from each study. The use of tables also aim to clarify the different contexts and themes that have been explored by the researchers, showing the procedures adopted to examine it, as well as their contributions on the subject.

**Table 2:** Emerging themes related to relational spirituality and quality of life for the last 10 years

References	Domain of QoL & Instrument	Meaning of RS & Instrument	Main Idea
Idler et al. [62].	Health status and functional ability, family and friendship networks, and psychological well-being *Instrument: 20-item Center for Epidemiological Studies Depression Scale.*	The idea of a connection to the sacred through religious rituals or experiences that serve as sources of strength and comfort in the last year of life. *Instrument: Designed variable for ratings of subjective religiosity*	QoL in the last year of life is positively related to subjective religiosity due to the social support that is gained in the process of associating with religious believers.
Saffari et al. [63].	Mobility, usual activities, self-care, pain/discomfort and anxiety/depression *Instrument: EQ-5D-3 L (which includes the EQ-5D assessing mobility, self-care, usual activities, pain/discomfort, anxiety/depression; and the Visual Analogue Scale (EQ-VAS) that allows respondents to rate their current health status from 0 to 100.*	Intrinsic religiosity and private religious activities that draw a sense of connection to the sacred. *Instrument: Spiritual Coping Strategies (SES) and The Duke University Religion Index (DUREL)*	Spiritual resources may contribute to better QoL and health status among haemodialysis patients.
Nagpal et al. [29].	Perceived QoL, behavioural competence, psychological status, and interpersonal environment *Instrument: The Quality of Life -Alzheimer’s Disease Scale (QoL-AD)*	Prayer, meditation, and subjective ratings of religiosity *Instrument: Designed variable for subjective ratings of religiosity (How religious or spiritual would you say you are?) answered on a Likert scale from 1 (not at all religious/spiritual) to 4 (very religious/ spiritual).*	The religiosity of a caregiver for an individual with Dementia may affect the perception of QoL of the individual they are looking after. In contrast, one’s own perception of spirituality does not guarantee QoL.
Miller et al. [30].	Emotions, confidence, self-esteem, physical health *Instrument: Heart disease specific QOL questionnaire*	Prayer or meditation, consequential religiosity for coping with personal problems, theological belief system, experiential religiosity pertaining to feeling of religious comfort. *Instruments: The Spiritual and Religious Concerns (SRC) questionnaire; The Religiosity Measure (RM); Religious coping activities scale.*	The distress following a cardiac event may require support from religious behaviour and spiritual beliefs. However, if there is no such support, as shown in the results, the authors argue that lower perceptions of QoL may trigger negative forms of religious coping and put the couples at risk of spiritual distress.
Nguyen et al. [75].	Functional status, physical health, and mental health *Instrument: Assessed indicators of physical HRQoL and mental HRQoL using the MEPS which includes the 12-item of the Medical Outcomes Study questionnaire (SF-12v2)*	Self-prayer *Instrument: Asked respondents if they used self-prayer as a form of complementary therapy within the past year. Responses to the item were combined to create a dichotomous variable reflecting the prayer and other five complementary therapies recognized by the US National Center of Complementary and Alternative Medicine (NCCAM), including alternative medical systems (*i.e.*, any use of acupuncture, Ayurveda, homeopathy, or naturopathy), biologically based therapies (*i.e.*, any use of chelation therapy, folk medicine, herb use, diet-based therapy, or megavitamin therapy in the past year),, manipulative and body-based methods (*i.e.*, any use of chiropractic and massage in the past year), mind-body medicine (*i.e.*, any use of biofeedback, relaxation techniques such as meditation, hypnosis, movement therapies such as yoga, or healing rituals in the past year),*	Prayer and having a sense of connection to the sacred may be a complementary health practice among older adults since it is used more than as any other alternative health therapy. However, this position may change over time since it is not necessarily associated to QoL.
Ai et al. [64].	Psychological functioning (behaviour coping, cognitive coping, levels of distress, anger coping, avoidant coping, depression), physical functioning (fatigue symptoms), social relationships (perceived social support). *Instruments: Short-term postoperative quality-of-life (SPQOL) (levels of distress, fatigue symptoms, levels of coping, and perceived social support) was measured by modifying several scales: 14-item Fatigue Scale, Center for Epidemiologic Studies Depression Scale (CES-D), Multidimensional Coping Scale, and the 12-item Multidimensional Scale of Perceived Social Support*	Coping by praying in private *Instrument: 3 items of the Using Private Prayer as a Means of Coping (Ai* et al.*, 2002) was used to assess prayer coping based on its appraisal (“Private prayer is important in my life”), efficacy (“Prayer does not help me to cope with difficulties and stress in my life”), and intention to use (*e.g.*, “I will use private prayer to cope with difficulties and stress associated with my cardiac surgery”)*	“Psychosocial factors may explain the potential role of using prayer for coping on short-term postoperative QoL” (p. 471).
Calvo et al. [70].	Physical health and overall QoL (i.e. emotional health, social well-being, and spiritual and financial aspects); psychological functioning (i.e., levels of anxiety, symptoms of depression, life satisfaction) *Instruments: McGill Quality of Life Questionnaire (MQoL); Satisfaction with Life Scale (SWLS); Zung Depression Scale (ZDS); Spielberger ‘s State and Trait Anxiety Inventory (STAI)*	Private/subjective religiosity *Instrument: 4-item Idler Index of Religiosity (IIR)*	The religiosity of caregivers of patients with ALS can be a helpful coping resource for negotiating their own QoL. Hence, “Health care professionals caring for ALS patients should consider that the needs of the caregivers include religious/spiritual concerns” (p.168).
Kashdan & Nezlek [72].	Psychological well-being, meaning in life, positive affect, self-esteem. *Instrument: Items were adapted from existing scales to assess self-esteem (Rosenberg Self-Esteem Scale), meaning in life (using two items,* e.g.*, “How meaningful did you feel your life was today?” “How much did you feel your life had purpose today?”), and responses to negative (nervous, embarrassed, upset, disappointed, bored, and sad) or positive (enthusiastic, excited, happy, calm, satisfied, and relaxed) affects as the conceptualisation of QoL*	Daily spirituality, Personal relationship with a power greater than one’s self, the spiritual part of one’s life. *Instrument: 22-item Spiritual Involvement and Beliefs Scale (SIBS-R) assessing Core Spirituality (*e.g.*, “I have a personal relationship with a power greater than myself”; “I solve my problems without using spiritual resources”)*	The study shows that spirituality is used as a coping strategy to deal with negative emotions. This is because the negative emotion experienced on 1 day is likely to predict increases in spirituality on the next day.
Nolan et al. [65].	Physical health, psychological health, social relationships, environmental health. *Instrument: World Health Organization Quality of Life–BREF (WHOQoLBREF)*	Religious coping, being connected to God through participating in prayer groups, meetings, and religious activities, meditation, spiritual reading, religious forgiveness, spiritual connection to God, benevolent religious reappraisal *Instrument: 14-item RCOPE consisting of the positive religious coping pattern (*e.g.*, religious forgiveness, seeking spiritual support, collaborative religious coping, spiritual connection, religious purification, and benevolent religious reappraisal) and the negative religious coping pattern (*e.g.*, spiritual discontent, reappraisal of God as punishing, interpersonal religious discontent, reappraisal of demonic powers, and reappraisal of God’s powers).*	Spirituality or religious coping in the form of community prayer services and devotion to God is an important factor that may have a major impact on the treatment of patients of African-American origin with Schizophrenia.
Bradshaw & Kent [22].	Psychological Well-Being *Instrument: three measures of psychological well-being: self-esteem, optimism, and life satisfaction*	Attachment to God through prayer *Instruments: Attachment to God Scale and frequency of prayer was based on a single item (How often do you pray by yourself?)*	A relationship with God makes prayer effective: the impact of prayer on one’s QoL depends on one’s perceived relationship (attachment) with God.
Sorensen et al. [66].	Psychological wellbeing, physical health symptoms, self-image, sexuality *Instrument: Psychological wellbeing in terms of life satisfaction was measured by the item: “Thinking about your life at the moment, would you say that you, by and large, are satisfied with life, or are you mostly dissatisfied?”, with response option dichotomised into ‘Dissatisfied with Life’ and ‘Satisfied with Life’. In addition, physical health was assessed using The EORTC breast cancer module (BR-23), prostate cancer module (PR-25), and colorectal cancer module (CR-29) which cover symptoms, self-image, sexuality, and specific complaints during the previous week.*	Seeking God’s Help. *Instrument: A sub-scale of the Instrument of Religious Coping (RCOPE) (*e.g.*, “I seek God’s help when I need strength and solace”) was dichotomized into two response options: ‘Seeking God’s Help’ and ‘Not Seeking God’s Help’.*	Cancer patients in Norway are not likely to seek God’s help to negotiate their QoL.
Yun et al. (2012).	Physical health, psychological health. *Instrument: European Organization for Research and Treatment of Cancer quality-of-life questionnaire core-30 (EORTC QLQ-C30) instrument*	Prayer therapy *Instrument: Investigated participants’ use of mind–body intervention (yoga, meditation, prayer therapy, music/dance therapy, art therapy, and horticultural therapy).*	Using mind-body interventions (e.g. prayer) may not be helpful for Korean cancer patients.
Levin [73].	Physical health *Instrument: Activities of Daily Living Scales and variables on indicators of physical health such as self-rated health, long-term health problems, activity limitation, diagnosed chronic diseases, physical symptoms.*	Frequency of prayer *Instrument: Designed variables for synagogue activities (*e.g.*, “Have you done any of these activities in the last month? Taken part in a religious organisation [church, synagogue, mosque,* etc.*])”, prayer (*e.g.*, “Thinking about the present, how often do you pray?”), and religious education (*e.g.*, “Have you been educated religiously by your parents?”)*	Religious involvement in the Synagogue is a much stronger predictor of better physical health than prayer, among Jewish people.
Moon & Kim [28]	Physical health status, mental health status, social relationships, and the environment. *Instrument: Geriatric quality of life-dementia (GQOL-D)*	Subjective religiosity. *Instrument: Duke religion index (DUREL)*	Subjective religiosity may account for the difference in QoL based on one’s religious background
Krumrei et al. [74]	Physical health *Instrument*: *Physical Component Summary score of the Short Form Health Survey (SF-12)*	Trust/mistrust in God and religious coping *Instrument*: *Brief Trust/Mistrust in God Scale; Jewish Religious Coping Scale (JCOPE); 2 items of the intrinsic religiosity subscale of the Duke Religion Index*	“Beliefs about the Divine activate coping strategies during times of distress, which in turn impact psychological health” (p.327). Highlights spirituality as having a clinical significance in mental health among Jews
Lee, Nezu, & Nezu [27].	Life satisfaction, Health worries, Financial worries, Medication worries, HIV mastery, Disclosure worries, Provider trust, and Sexual functioning *Instrument:* The 34-item HIV/AIDS-targeted quality of life (HAT-QoL)	Spiritual connection for religious coping *Instrument:* Religious Coping Scale (RCOPE),	Religion is an important resource for people living with HIV/AIDS
Currier et al. [68].	Physical health, psychological health, social and environmental QoL*.* *Instrument: World Health Organization’s Quality of Life Scale (WHOQOL-bref)*	Spiritual functioning in terms of forgiveness of self, others, and God. *Instrument: Brief Multidimensional Measure of Religiousness and Spirituality (BMMRS)*	Forgiveness is an important factor to consider when modelling for spirituality and QoL.
Canada et al. [69].	Mental functioning, physical functioning, spiritual functioning. *Instrument: SF-36 Physical and Mental Health Summary Scales*	Feelings of comfort and strength *Instrument: Functional Assessment of Chronic Illness Therapy–Spiritual Well-being Scale*	Faith makes an important contribution to the QoL of cancer survivors.
Krause et al. [76].	Physical health *Instrument: Physical health was measured using the Physical Component Summary score of the Short Form Health Survey (SF-12).* *Depressive symptoms were also assessed as an aspect of psychological QoL functioning using a short form of the Center for Epidemiologic Studies Depression Scale (CES-D).*	God image representations; intrinsic religiosity; trust in God *Instrument: 6-item Brief Trust/Mistrust in God Scale (*e.g.*, “God cares about my deepest concerns”; “God hates me”); 16-item Jewish Religious Coping Scale (JCOPE) (*e.g.*, “I try to see how God may be trying to teach me something,” “I question my religious beliefs, faith and practices,” and “I look for a stronger connection with God”).* *Intrinsic religiosity was measured using three items from the Duke Religion Index which are also present in the Religious Orientation Scale and the Hoge Intrinsic Religion Scale (*e.g.*, “My religious beliefs are what really lie behind my whole approach to life,” “In my life, I experience the presence of the Divine (*i.e.*, God),” and “I try hard to carry my religion over in to all other dealings in life”).*	Spiritual/social support from fellow members of a church influences one’s perception of God as benevolent and gratitude to God, which in turn leads to better health.
Rohani et al. [71].	Physical health *Instrument: European Organization for Research and Treatment of Cancer QLQ-C30; Sense of Coherence (SOC) scale*	Spiritual behaviours and views that are developed through private prayer or meditation *Instrument: Spiritual Perspective Scale; the Brief Religious Coping Scale*	Spirituality is not associated with QoL for Iranian women with breast cancer.

The fourth element of the 5-step guideline for IR involved reviewing selected studies in order to identify emerging themes that may help in conceptualizing the association between RS and QoL, as shown in Table 2. Themes summarized here help to identify areas for future studies and disclose relevant potential questions central to RS and QoL. Finally, emerging themes from our review were organized and critically analysed in the discussion section in relation to addressing the research problem on claims about the association between RS and QoL. Analysing study themes generated from reviewing diverse research output is expected to account for the integrated framework in terms of understanding the complexity of RS and QoL research. In conducting an IR, the authors addressed questions which related to existent knowledge regarding about the links between RS and QoL, the quality and complexity associated with these links, and steps for future research into investigating the effects of RS on QoL [58].

## Results

All selected studies have been summarised in Table 1, showing evidence of positive (***), indirect (#*), negative association (**), and lack of association (#) between RS and QoL. A total of 132,053 participants were involved across the 20 selected studies. In particular, 70% of the studies were conducted with participants suffering from one form of mental or physical health-related challenge which included: 499 terminally ill elderly persons [62], 362 Muslim patients undergoing haemodialysis [63], 111 individuals with dementia [29], 44 couples (dyads) following a first-time cardiac event [30], 294 patients following an open-heart surgery [64], 63 outpatients with schizophrenia [65], 1024 older American adults [22], 8344 Norwegian cancer patients [66], 481 terminally ill cancer patients [67], 198 HIV/AIDS infected patients [27], 678 military veterans with PTSD [68], 8405 cancer survivors [69], 75 ALS patients and their caregivers (dyads) [70], and 162 Iranian women with breast cancer [71]. The remaining 30% were conducted with other study populations, including 87 college students [72], 1287 older Jews in Europe [73], 208 Jewish men and women [74], 274 older Koreans living alone in Chuncheon [28], 107, 683 older adults in a national health survey [75], and 1774 American adults [76].

All the 20 studies selected for the review employed quantitative research designs, while one study adopted a mixed-methods design (i.e., [29]). With the exception of a few prospective cohort studies (e.g., [22, 62, 67, 75]), for the most part a cross-sectional design was used.

### Summarizing the frequency of association between RS and QoL

#### Positive association between RS and QoL

As shown in Table 1, of the 20 articles used for the predictive analyses outcomes, 12 (60%) of the studies with 12,917 participants reported positive associations between RS and QoL (e.g., [27, 28, 62–65, 68–70, 72, 74, 76]). Participants in these studies reported on the main effects of RS on their QoL, showing correlations between measures of RS and QoL. For example, Saffari et al. [63] found that spiritual resources accounted for substantial variance to better QoL and health; Idler et al. [62] reported that individuals with stronger religious attachment were more likely to have better QoL and fewer depressive feelings. In another report (e.g., [64]), RS, through adaptive faith-based coping mechanism such as prayer, was positively associated with psychological functioning among cardiac patients; this relationship was also strengthened by perceived social support from family, friends, and significant others at the time of participant’s surgery. The study showed the indirect influence of using prayer for coping on short-term QoL and positions prayer to functions as “the mediation of cognitive coping and perceived social support”. Calvo et al. [70] reported that QoL and life satisfaction of the caregivers of patients with ALS was associated with aspects of RS. Kashdan and Nezlek [72] reported a significant positive relationship between daily RS and psychological functioning (i.e., self-esteem, meaning in life, and positive affect).

Evidence shows that RS was associated with greater QoL and psychological health in a study [65] with mainly African American Christians in South-eastern United States (commonly identified as the Bible Belt region), where 98% of the respondents were involved in various aspects of RS that include religious activities such as prayer meetings, being connected to a religious community, praying once a day, meditation, and spiritual reading; as well as religious coping activities such as practising forgiveness, seeking spiritual support, collaborative religious coping, spiritual connection with God, religious purification, and benevolent religious reappraisals. Krumrei et al. [74] report a positive correlation between measures of physical health and RS in terms of ‘trust in God’ in both the adjusted and unadjusted models, and an inverse association between physical health and mistrust in God (i.e., didn’t trust God). RS accounted for the variance in depression and QoL among older Christian Koreans, with results showing significant associations between measures of RS and QoL [28]. However, this was not the case for Buddhist older Koreans who seem to attend religious meetings less regularly than the Christian respondents, based on the observations of the authors. Currier et al. [68] report on the salutary effects of spiritual functioning in terms of forgiveness of self and others on QoL outomes, noting that “Higher levels of spiritual functioning were associated with fewer forgiveness problems among Veterans, and their propensity to forgive self and others was also concurrently linked with QOL” (p.175). Other studies further show evidence of a positive association between a person’s faith experience and mental and physical functioning (e.g., [69]), and between spiritual behaviours and views developed through prayer and increases in QoL (e.g., [71]).

All these 12 studies make a case for the effects of RS on QoL and provide evidence to show that RS accounted for the variation in QoL and health status, particularly among older adults [28, 62, 64], military Veterans [68], cancer patients [69], persons with HIV/AIDS [27], Jewish men and women [74], lonely elderly Koreans [28], outpatients with schizophrenia [65], college students [72], patients with Amyotrophic Lateral Sclerosis [70], patients recovering from heart surgery [64], patients undergoing haemodialysis [63], and patients with posttraumatic stress disorder [68].

### Indirect association between RS and QoL

The study with individuals with dementia (IWD) coded in the ‘no association’ (#) index in Table 1 (e.g., [29]) reported a lack of association between RS and QoL, but recorded an indirect relationship when a third party is involved (IWD caregivers). Nagpel and colleagues concluded that the spirituality of a caregiver of IWD may influence perceptions of QoL among IWD. Another study coded in the ‘no association’ category (e.g., [22]) reveals that prayer (as an activity of RS) is not directly associated with improvement in QoL, except when moderated by attachment to God (having a relationship with God). The study by Bradshaw and Kent suggests that without a relationship with God the frequency of prayer is ineffective in terms of improvements in psychological well-being. These indirect associations reflect the complexity of the positive relationship between RS and QoL, showing that the effects of RS on QoL may be dependent on other factors and activities of RS that translate into better health outcomes. Our findings support the salutary effects of RS on QoL, be it through direct or indirect associations, as shown in Table 1.

### Negative association between RS and QoL

As shown in Table 1, three (15%) studies with 10,112 participants reported inverse associations between RS and QoL [66, 67, 73]. Sorensen et al. [66] report that the prevalence of RS in terms of ‘seeking God’s help’ was negatively associated with sexual QoL, although this relationship was not retained when controlled for other factors such as gender, age, anxiety, neuroticism, extraversion, follow-up time, daily smoking, infrequent exercise, negative outlook, and positive outlook.

Yun et al. [67] report that the RS of terminally ill Korean cancer patients showed a significantly worse survival of insomnia and change in health-related QoL subscales. Yun and colleagues examined the health outcomes of Complementary and Alternative Medicine (CAM) among users and non-users. In comparing CAM users and non-CAM users, they found that CAM users did not have better survival ratio than nonusers. Prayer was identified as an aspect of CAM, but results showed worst insomnia survival and cognitive functioning for terminally ill cancer patients who use it as a religious coping behaviour, compared to that of nonusers. Yun et al. concluded that CAM use is associated to worst health outcomes, even though some patients often used CAM in the hope that it will cure their illness. Yun et al. also pointed to the limitations of the study as the possible reasons for the results. First, they note that it is difficult to draw firm conclusions from the results due to the nonrandomized design used for data collection. Secondly, they reasoned that due to the small sample size used for the analysis of CAM subcategories (e.g. meditation, prayer therapy, music therapy, art therapy, yoga, horticultural therapy), the statistical power might have been too weak to detect benefits of CAM use.

Levin [73] reports that RS in terms of prayer was negatively related to physical health QoL among Jewish respondents age 50 and above [73]. In the unadjusted model, prayer was negatively correlated to self-rated health, but positively associated with physical symptoms, poor physical functioning, and activity limitation. Adjusting for covariates (e.g., age group, gender differences, education background, relationship status, and birth place) and mediators (e.g., drinking, smoking, and help received from outside the home), the frequency of prayer retained its negative association with self-rated health, and positively correlated with physical symptoms, poor physical functioning, and activity limitation. In contrast, Synagogue participation had a better physical health outcome for Jewish respondents who seem to have a strong attachment to the Synagogue. According to Levin, the dynamics of Jewish spirituality may explain the negative relationship between prayer and physical health since it comparatively has to do with integration into a synagogue community than it does with using prayer as a coping mechanism. As Levin suggests, both findings may in part result from the effects of preventing Synagogue participation and focusing on using prayer as a religious coping behaviour, since “certain expression of religiousness may decline with poor health (e.g., synagogue attendance) and others may increase as a coping response (e.g., prayer)” ([73]: 598).

### No association between RS and QoL

As shown in Table 1, the results reveal that five (25%) studies showed no direct association between aspects of RS and outcomes of QoL [22, 29, 30, 71, 75], even though two of these studies (i.e., [22, 29]) later reported an indirect association between RS and QoL via psychosocial factors such as secure attachment to God and perceived social support from caregivers who have a relationship with God. In addition, participants in three of these studies had serious illnesses, ranging from dementia [29], cardiac arrest [30], and breast cancer [71], while data from the two other studies [22, 75] were also retrieved from health-related contexts. This connotes that the lack of association in the illness context between RS and physical health-related QoL may be due to the salutary effects of RS, which provide comfort but do not change the physical status of the patients. In contrast, RS was strongly correlated with psychological functioning, as shown in other studies (e.g., [62, 64]).

It is possible that the different results in terms of the positive, negative, indirect or not detected associations between RS and QoL could be due to conceptualisation and operationalisation of key variables and different study designs (see Table 2). For example, most of the ‘no association’ studies used health-specific QoL measures to assess the physical health domain of participants. Nepgal et al. [29] examined QoL using the Quality of Life Alzheimer’s Disease Scale (QoL-AD); Miller et al. [30] used a Heart-disease specific QoL questionnaire; the same approach was taken by Nguyen et al. [75]. Studies that showed ‘positive association’ (e.g., [27, 28, 63, 68, 74]) mostly employed cross-sectional designs which make it impossible to make firm claims about causality in the IR. However, it is also possible that the estimated effects of aspects of RS on outcomes of QoL are conditional upon each other, e.g., that the effects of RS that are perceived as both positive (e.g., seeking spiritual support) and negative (e.g., spiritual discontent) religious coping emotions and behaviours could be especially consequential for QoL outcomes. These complex patterns warrant further investigation.

### Identifying emerging themes

This section identifies themes used in conceptualising RS and QoL. First, we examine themes used in all the studies for examining RS as a domain of QoL that relate to spiritual functioning and a relational dynamic in terms of attachment with a symbolic sacred object or figure via implicit relational conduits. Second, we further identify themes used for the conceptualisation of QoL in all the studies as aspects of individual needs satisfaction with key areas of life that contribute to overall wellbeing.

### Meanings of RS

Although the study data contain different definitions of RS as shown in Table 2, it still conveys similar relational ideas of attachment to the sacred (e.g., [22, 27, 76]). The meanings attached to these relational experiences serve as sources of strength and comfort in one’s life [62], taking the form of private spiritual activities that draw one closer to the sacred [63], and a personal relationship with a power greater than one’s self or the spiritual part of one’s life [22, 72]. RS was also commonly interpreted as ‘subjective religiosity’ [28, 29, 70], ‘intrinsic religiosity’ [63], ‘experiential and consequential religiosity’ [30], ‘spiritual connection’ [27], ‘attachment to God’ [22], ‘spiritual functioning’ [68], ‘God image representations’ [76], spiritual behaviours and views [71], and as an aspect of spiritual functioning that has to do with forgiveness of self and others [68].

In addition, most of the studies referred to RS as a frequency of ‘prayer’ or as a form of spiritual transcendence that is achieved through private prayer (e.g., [29, 30, 64, 67, 71, 73, 75]). However, Bradshaw and Kent [22] interpreted RS differently as an attachment to God experience achieved through prayer. Nolan et al. [65] saw RS as a connection to the sacred that is facilitated through the context of a faith community. This is similar to the findings of Nagpal et al. [29] and Ai et al. [64] who also stressed the role played by psychosocial factors in terms of perceived social support and community prayer services in their conceptualisation of RS. One study [69] had a vague definition of RS as ‘feelings of comfort and strength’, even though this generally suggests an idea of spiritual functioning.

Levin [73] presents a different understanding of RS which considers the intersection of religion and place as a conceptualisation of Jewish RS, which is formed within the context of the Synagogue experience. Different RS themes and perspectives were identified in the selected studies. As shown in Table 2, the results from these studies are likely to be influenced by the different conceptualisation used by respective researchers. In addition, the results from the studies may also be linked to an operationalisation problem since RS was assessed based on a variety of designs. For example, some studies measured the RS construct using unstandardized subjective ratings of religiosity (e.g., [29, 62, 75]), while other studies (e.g., [22, 30, 63, 74]) measured RS using psychometric assessments such as The Spiritual and Religious Concerns questionnaire, The Religiosity Measure, Religious coping Activities Scale, Attachment to God Scale, Brief Trust/Mistrust in God Scale, Jewish Religious Coping Scale (JCOPE), Duke Religion Index.

### Domains of QoL

Results on aspects of QoL reported in all the studies are presented in Table 2. Aspects of QoL assessed in some of the studies were not clearly defined, even though the concepts used to describe QoL corroborate our theoretical assumptions. All the reviewed studies assessed several QoL domains that include psychological, physical, social, and spiritual functioning, while few studies seem to integrate other health-related factors as aspects of QoL. For example, we observed that a number of studies conceptualised QoL in terms of a single indicator such as depressive symptoms (e.g., [62, 63, 70, 76]), fatigue symptoms (e.g., [64]), and levels of anxiety (e.g., [63]). As expected, these unconventional definitions led to using instruments that are not necessarily designed for measuring QoL, such as the Fatigue Scale, Center for Epidemiologic Studies Depression Scale, Medical Outcomes Study questionnaire. However, two QoL aspects (i.e., psychological functioning and physical health) are the most researched domain of QoL in medical-health research, as all the studies assessed either one or both aspects.

Interestingly, Calvo et al. [70] took a more holistic approach in their examination of QoL, as they investigated physical health, psychological functioning, and other aspects of QoL that are related to social well-being, spiritual functioning, and financial aspects among Amyotrophic Lateral Sclerosis patients. Canada et al. [69] integrated aspects of mental functioning, physical functioning, and spiritual functioning in their study of faith and QoL of cancer survivors. As shown in Table 2, most of the QoL scales used in selected studies were modified, based on specific cases of interest, such as the HIV/AIDS-Targeted QoL (e.g., [27]), Geriatric QoL-dementia (GQOL-D) [28], QoL – Alzheimer’s Disease Scale (e.g., [29]), Heart-disease Specific QoL Scale (e.g., [30]), McGill QoL Questionnaire (e.g., [70]), Physical and Mental Health Summary Scales (e.g., [69]), World Health Organisation QoL Scale (WHOQoL-BREF) (e.g., Currier et al. [68], and the European Organization for Research and Treatment of Cancer QoL Questionnaire (e.g., [67]). Other assessment techniques were based on designing categorical variables (e.g., nominal, dichotomous, or ordinal) on subjective ratings of questions connected to aspects of health-related QoL that may have health implications for the participants (e.g., [66, 72, 73]).

## Discussion

Among the most important findings has been the fairly consistent positive correlation between RS and QoL. This finding contrasts with the classical thesis which underestimates the continuing social influence of religion on healthcare, as religion can sometime be perceived as involving moral restrictions that contribute to guilt and, hence, negative emotions that predispose to depression and poor QoL [57]. As presented in Tables 1 and 2, our review of the association between QoL and RS reveals the following:

I) There is good evidence that RS is associated with better outcomes of QoL in the areas of physical health, psychological wellbeing, social relationship quality, and spiritual functioning.
II) The effects of RS on QoL may take the form of direct and/or indirect associations, depending on the psychosocial factors (e.g., perceived social support by fellow members of the church, a religious caregiver, or group prayer services) and contexts (e.g., illness, secular, or religious) involved.
III) The effects of RS on QoL may depend on the right application of meaning in terms of how it is defined (conceptualisation) and measured (operationalisation).
IV) RS coping activities and beliefs (e.g., trusting in God, prayer, positive spiritual connections, or forgiving self and others) activate coping strategies during times of distress, leading to better outcomes of QoL.

These findings highlight the significance of RS as an important component for negotiating day-to-day QoL. While most of the studies identify RS as a form of coping strategy with health benefits, in 75% of the reviewed studies (*** and #*), others showed the role of perceived social support in enhancing the effects of RS on QoL via caregivers who are religious [29], being part of a faith community [64], or seeking spiritual support and collaborative religious coping through community prayer services [65]. Psychosocial activities that include practices of RS became a helpful coping recourse for achieving better QoL and played a major role in the way participants experienced spirituality and negotiated their QoL.

The role of prayer in moderating the effects of RS is also noteworthy in the IR (e.g. [22, 64, 75]). Prayer was often used by the participants as a spiritual conduit for assessing the sacred and an important aspect of RS, with studies referring to ‘prayer’ as a form of religious coping behaviour. Overall, prayer was the most commonly used aspect of religious coping behaviour among older adults, and was positively associated with cognitive, behavioural and psychological changes.

However, religious coping behaviour may not be used as a coping therapy in every context, as demonstrated in studies conducted within the illness context (e.g., Miller et al.; [29, 30, 71, 75]), secular context (e.g., [66]), and religious context (e.g., [28, 73]), which highlight this shift in perspective. First, we see this shift in health-specific studies (except for [74]) reporting on a variety of patients (e.g., dementia, cardiac arrest, and breast cancer patients), which found that RS was not related to physical health QoL. This suggests that even though RS may have provided comfort, it does not necessarily change the physical health status of the patients. Second, we also found that the same applies for participants in the secular Norwegian context (e.g., [66]) whose RS did not translate to better health outcomes.

Third, another study (e.g., [28]) also support that the effects of RS on QoL is context-driven, and may be different depending on individual religious background, or how RS is practiced as in the case of Jewish participants (i.e., [73]) whose integration into the Synagogue community was a significant predictor of better physical health than their prayer life. Moon and Kim reported that spirituality accounted for the variance in QoL among Korean Protestants and Catholics, but not for the Buddhist sample. One interpretation given for this result was that “Buddhists usually attend religious meetings less regularly than Protestant or Catholics” ([28]: 298), and that Buddhists had lower RS outcomes than Protestants and Catholics. These different perspectives suggest varying accounts of the salutary effects of RS on QoL, which seem to vary based on the individual’s social context. In other words, even though RS may have provided symbolic beneficial experiences (e.g., a sense of meaning, social support, and spiritual connection) that are good for negotiating QoL, these health benefits can also be because of other sources such as the social context that affect the spiritual role. To better understand the role of RS on health outcomes, we need to consider the “ways in which culture influences religion’s expression of the spiritual” ([77]: S54).

This IR has shown the discrepancies in themes used to describe RS and QoL over the last 10 years. Table 2 shows the different themes associated with all the studies, although the various conceptualisations still point to some sort of relational dynamics that involve attachment to the sacred and aspects of spiritual functioning. Of the 20 studies identified, none exclusively used the term “relational spirituality”, even though the conceptualisations bear semblance with our definitions of RS. The specific terms used in describing RS in most of the studies are “religion”, “religiosity”, “faith”, “spirituality”, “complementary therapy”, “private prayer”, “religious coping”, “attachment to God”, “seeking God’s help”, “Jewish spirituality”, and “images of God.” In using these terms, most of the studies failed to clearly define RS as a relational dynamic that deals with attachment to the sacred. Instead, they used measures and categorical variables that assess aspects of RS that involve intrinsic aspects, such as meditation, prayer, and attachment to a higher power. Conceptually, RS continues to change while retaining the relational dynamic of its tradition.

At present, an attachment-based model of RS, though achieved through prayer and other religious-related activities with others and positive perceptions of the divine, seems to be the overriding theme in studies examining RS and QoL over the past 10 years. God image representations and trust/mistrust in God as seen in the works of Krause et al. [76] and Krumrai et al. [74] also re-emphasize similar relational perspectives, along with other studies which demonstrate a similar attachment-religion connection. These conceptualisations are likely because of the operation of Bowlby’s [43] proposed internal working models of attachment which, even though developed during the early years of life, remain active in one’s lifespan attachment development [44] and determine the extent to which a potential object of attachment is experienced, even in the RS context (e.g., [13, 16, 37]).

Long [78] found a significant correlation between measures of attachment to God RS, parental bonding, and religious coping. The ability to regulate emotional states in RS contexts due to the internal working model of the attachment system may be the very key to unfolding spiritual coping model for negotiating QoL when drawn to the sacred. The direct and/or indirect associations with QoL suggest the activation of a spiritual coping model that helps in relational processes and in dealing with stressful life situations, based on the connection to what is transcendent. The idea of the transcendent can take different directions, as shown in our IR as spiritual functioning, forgiving self, forging a meaningful relationship with others, and building a sense of attachment to God. This framework of a spiritual coping model is more active in the third aspect in terms of the association between RS and QoL.

The limitations across these 20 studies include the following: mostly focused on older adults with younger people unrepresented; mostly quantitative design which lacks in-depth perspectives capture; mostly cross-sectional studies with limited causality examination; lack of QoL specific measures; and the use of categorical rather than continuous operationalization of RS and QoL.

Although study findings show evidence of the links between RS and QoL in medical-health research for the last decade, we were unable to retrieve some studies related to RS and QoL during our initial literature search due to indexing problems and date of publication. After conducting a manual search of related articles reporting on the links between RS and QoL, we found some related studies (e.g., [16, 37, 79–83]), but were not able to report them because they were either not indexed in the selected databases (i.e., PubMed, ScienceDirect, and PsycINFO), using a different conceptualisation of QoL (e.g., levels of emotional distress), or were published after our deadline of literature search (i.e., March 2017). However, the results from these “missing” studies also support the salutary effects of an attachment-based model of RS on levels of psychological wellbeing. We are confident that the studies selected for this IR are good representatives of relevant studies published within the past 10 years demonstrating how RS has been perceived/positioned in relation to QoL.

## Conclusion

The findings from this review have broad implications for the role of RS in relation to QoL in medical-health contexts. Existing models of RS coping are better understood by integrating spiritually-based perspectives that include identifying aspects of faith and individual life that may be understood as a contextual ‘biopsychosocial spirituality’ [64]. Greater awareness of the importance of RS among healthcare professionals may improve cultural competence in healthcare services and community support in addressing patient’s spirituality, and strengthen collaborative relationship between healthcare and faith-based organizations. Further research on the relation between RS and QoL should be conducted across different population and health contexts.

## Footnotes

1 Health Science Descriptors

2 Medical Subject Headings

3 Table 1 explores and summarizes the general categories of the articles in terms of the study characteristics.

4 Table 2 contains themes and meanings that emerge from the studies for describing the link between RS and QoL.
